# Editorial: Development of Healthy and Nutritious Cereals: Recent Insights on Molecular Advances in Breeding

**DOI:** 10.3389/fgene.2021.635006

**Published:** 2021-03-18

**Authors:** B. P. Mallikarjuna Swamy, Balram Marathi, Ana I. F. Ribeiro-Barros, Felipe Klein Ricachenevsky

**Affiliations:** ^1^International Rice Research Institute, Los Baños, Philippines; ^2^Department of Genetics and Plant Breeding, Professor Jayashankar Telangana State Agricultural University, Hyderabad, India; ^3^Forest Research Centre (CEF), Instituto Superior de Agronomia, Universidade de Lisboa, Lisbon, Portugal; ^4^Departamento de Botânica, Instituto de Biociências, Universidade Federal do Rio Grande do Sul, Porto Alegre, Brazil; ^5^Programa de Pós-Graduação em Biologia Celular e Molecular, Centro de Biotecnologia, Universidade Federal do Rio Grande do Sul, Porto Alegre, Brazil

**Keywords:** cereals, nutrition, breeding, omics, genome editing, biofortification

Worldwide more than 2 billion people are affected by micronutrient deficiencies and most of them are residing in the developing countries of Asia, Africa and Latin America (Kennedy et al., 2002). Malnutrition is linked with heavy dependence on monotonous cereal staples without much dietary diversification or nutrient supplementation. Even though significant efforts have been made over the last six decades to improve production and productivity in most food crops, it lacked associated nutritional improvement (Bouis and Welch, 2010). So, the modern varieties do not have enough variability for several nutrients, making poor rural populations vulnerable to micronutrient deficiencies. More than two dozen mineral elements, vitamins, antioxidants, and health beneficial compounds must be supplied in optimal quantities daily for normal growth and development of humans. Biofortification of cereals with elevated levels of essential micronutrients, vitamins, and reduced levels of toxic elements help to address malnutrition and is a cost-effective approach in reaching target groups, especially rural populations (Bouis and Saltman, 2017). The sustainable development goals and the Lancet Commission Report have emphasized the need for promoting nutritious diets to eradicate malnutrition (Willet et al., 2019; https://sustainabledevelopment.un.org). Among these, deficiencies of iron (Fe), zinc (Zn), and vitamin A are major global health problems. As successful examples, one high Fe rice and several high Zn rice varieties have been successfully released for commercial cultivation (Palanog et al., 2019).

Presently we have a better understanding of the genetic, physiological, and molecular basis, as well as the influence of environmental factors on nutrients accumulation in cereal grains (Swamy et al., 2016; Garcia-Oliveira et al., 2018; Ludwig and Slamet-Loedin, 2019). However, there is a need to integrate our understanding to achieve the goals of biofortification and review the current progress and the prospects for nutritious crops. In this Research Topic, we selected manuscripts on various aspects of nutritional improvement in cereals. Fourteen articles published in our special editorial topic, five of them provided updated review of cereals nutritional enhancement, and nine of them were original research articles on understanding the molecular basis of different grain nutrients and grain quality traits in cereals.

Focusing on improving grain protein quality, Chandran et al. successfully pyramided Lysine, Tryptophan, and Provitamin A into Maize varieties based on *opaque-2 and* β*-carotene* through marker assisted selection (MAS). The improved lines possessed high lysine, tryptophan, and β-carotene content, but they had only slight yield reduction. Even though these lines can be used as genetic resources for maize improvement, they are not yet commercially viable, since successful biofortified crops should have similar or even higher yield along with the other desirable traits. Also aiming at improving protein content in rice, Jang et al. identified multiple genomic regions responsible for amino acid content (AAC) and protein content (PC). They identified two novel *loci* qAAC6.1 and qAAC7.1 and several transgressive segregants for both traits. These loci can be used for quantitative trait loci (QTL) pyramiding programs to develop rice lines with high protein content.

It is quite interesting to note pleotropic effect of heading-date genes on protein content of rice. Xie et al. reported that in three nearly isogenic lines (NIL), the rice florigen genes RFT1 have a strong negative effect on the amino acids content governed by the *Zhenshan97* allele with the genomic region consisting of 14 QTLs located in proximity to Hd3a. Bhuvaneswari et al. characterized 93 aromatic Chakhao rice germplasm from Manipur province of India. Wider variations were observed for the agro-morphological, grain quality and nutraceutical traits. The total anthocyanin content ranged from 29.8 to 275.8 mg.100g^−1^ DW, while total phenolics ranged from 66.5 to 700.3 mg GAE.100g^−1^ DW. The germplasm with higher levels of anthocyanin compounds such as cyanidin-3-O-glucoside (C3G) and peonidin-3-O-glucoside (P3G) are useful for improving the antioxidant properties in rice.

Focusing on micronutrient biofortification, Ashokkumar et al. comprehensively reviewed recent advances in breeding for improved folate, provitamin A, and carotenoids content in rice, wheat, maize, and pearl millet. They discussed in detail the genetic variation, trait discovery, genes/QTL identification for nutritional traits and their introgressions into elite genetic backgrounds. Prasanna et al. carried out a detailed global analysis of molecular breeding for nutritional improvement in maize, a species where systematic efforts have been made to develop and deploy cultivars biofortified with quality protein maize (QPM), provitamin A, and kernel zinc. The limited germplasm characterization, lack of genetic variability, and diagnostic markers for some of the mineral elements is a constraint for breeding. Broadening the genetic base through exploitation of landraces and wild species, use of genomics technologies, market-driven breeding strategies, strengthening of seed systems, and collaborative interdisciplinary efforts were emphasized. Genetic Engineering (GE) and Genome Editing (GEd) technologies are the way forward for improving the traits with no variability and to achieve the target levels of multiple nutrients in cereals.

Babu et al. characterized 40 rice genotypes for agronomic, yield and micronutrient traits. They identified stable high Zn donor lines and genome wide association analysis resulted in identification three *loci* on chromosomes 3 and 7, which were linked to new, uncharacterized putative candidate genes. Bollinedi et al. identified 18 novel marker-trait associations (MTAs) for grain Fe and Zn in brown and milled rice using 192 Indian rice germplasm accessions and found strong association between Zn concentrations in brown and milled rice. Fe concentration in brown rice, however, was not associated with Fe concentration in milled rice, highlighting the need for enriching the Fe concentration of rice endosperm. They have also identified four accessions with grain Zn concentration in milled rice with >28 mg/kg and one accession (IC-2127) with >12 mg/kg Fe, a target set by the HarvestPlus program for rice biofortification which will be used to develop high Fe and Zn varieties.

Focusing on biofortification of not-so-typical grains, Bekkering and Tian provide an overview of how other cereals and non-grass pseudo-cereals can contribute to biofortification. The monocots species, broomcorn millet (*Panicum miliaceum* L.), canary seed (*Phalaris canariensis* L.), teff [*Eragrostis tef* (Zuccagni) Trotter], and the pseudo-cereals (i.e., seeds similar to cereals, but not from the Poaceae family), amaranth (*Amaranthus* spp.), buckwheat (*Fagopyrum esculentum* Moench.), chia (*Salvia hispanica* L.), and quinoa (*Chenopodium quinoa* Willd), are richer in protein and lipids, and have lower starch compared to the major staples. Other characteristics include a more balanced protein composition, higher levels of micronutrients and vitamins. Authors delineate possible avenues in which we should invest to improve and fully utilize these species to provide nutritious grains for consumption, from marketing to breeding to genomics/functional genetics.

In the same line, Rodríguez et al., provided an extensive review on finger millet (*Eleusine coracana*) and foxtail millet (*Setaria italica*) and the pseudocereals quinoa, amaranth and buckwheat. These species can contribute to produce healthy grains, especially in harsh, stressful environments, for which these plants tend to be more resilient. Authors compare nutritional profiles, processes to increase bioavailability of nutrients and decrease anti-nutrients, and thoroughly review the current status of genome resources and molecular markers available to drive the efforts to improve these species, allowing their use in marginal lands to produce nutritious food to combat hidden hunger. Renganathan et al. focus on barnyard millet from the genus *Echinochloa*, including the cultivated Indian barnyard millet (*Echinochloa frumentacea*), Japanese barnyard millet (*Echinochloa esculenta*) and other wild species, which are used for human consumption and livestock feed, and also show tolerance to multiple stresses. The taxonomy, morphological and genetic diversity, genomic and genetic resources, and the potential of these species to contribute for food security and human nutrition are discussed. Altogether, these reviews are an excellent starting point for the biofortification community to launch efforts into including new species in our toolbox to increase human access to sufficient nutrients.

From a genomic perspective, Butardo et al. addressed the structural, regulatory and nutrition roles of Starch Synthase IIa in *O. sativa* ssp. *japonica* rice endosperm, highlighting the importance of this key enzyme in seed morphology, starch granules size and distribution, amylopectin structure, amylose content and glycemic index. Using a different approach, Kishor et al. used whole-genome next generation sequencing to characterize traditional varieties of basmati rice and identified millions of SNP markers useful for genetic analysis. Finally, Bhuvaneswari et al., combined genomics and metabolomics to bring forward the nutraceutical importance of aromatic glutinous rice through the characterization of a set of 93 landraces for their agro-morphological traits, grain pigmentation, antioxidant properties, and molecular genetic variation. Altogether these three papers provide a solid platform of molecular markers for taxonomy, breeding and conservation programs.

Our Research Topic combines different approaches on biofortification and provides a comprehensive collection of the efforts to improve grain nutrient quality and to increase human nutrition.

## Author Contributions

All the authors equally contributed to prepare this editorial manuscript.

## Conflict of Interest

The authors declare that the research was conducted in the absence of any commercial or financial relationships that could be construed as a potential conflict of interest.
